# Human enteric nervous system progenitor transplantation improves functional responses in Hirschsprung disease patient-derived tissue

**DOI:** 10.1136/gutjnl-2023-331532

**Published:** 2024-05-30

**Authors:** Benjamin Jevans, Fay Cooper, Yuliia Fatieieva, Antigoni Gogolou, Yi-Ning Kang, Restuadi Restuadi, Dale Moulding, Pieter Vanden Berghe, Igor Adameyko, Nikhil Thapar, Peter W Andrews, Paolo De Coppi, Anestis Tsakiridis, Conor J McCann

**Affiliations:** 1 Stem Cells and Regenerative Medicine, UCL GOS Institute of Child Health, London, UK; 2 NIHR Great Ormond Street Hospital Biomedical Research Centre, London, UK; 3 School of Biosciences, The University of Sheffield, Sheffield, UK; 4 Neuroscience Institute, The University of Sheffield, Sheffield, UK; 5 Department of Neuroimmunology, Centre for Brain Research, Medical University of Vienna, Wien, Austria; 6 Laboratory for Enteric NeuroScience (LENS), Translational Research Centre for Gastrointestinal Disorders (TARGID), Katholieke Universiteit Leuven, Leuven, Belgium; 7 Cell and Tissue Imaging Cluster (CIC), Katholieke Universiteit Leuven, Leuven, Belgium; 8 Department of Physiology and Pharmacology, Karolinska Institute, Stockholm, Sweden; 9 Gastroenterology, Hepatology and Liver Transplant, Queensland Children’s Hospital UQ Faculty, South Brisbane, Queensland, Australia; 10 Specialist Neonatal and Paediatric Surgery Unit, Great Ormond Street Hospital, London, UK

**Keywords:** ENTERIC NERVOUS SYSTEM, STEM CELLS, HIRSCHSPRUNG'S DISEASE, GASTROINTESTINAL SURGERY

## Abstract

**Objective:**

Hirschsprung disease (HSCR) is a severe congenital disorder affecting 1:5000 live births. HSCR results from the failure of enteric nervous system (ENS) progenitors to fully colonise the gastrointestinal tract during embryonic development. This leads to aganglionosis in the distal bowel, resulting in disrupted motor activity and impaired peristalsis. Currently, the only viable treatment option is surgical resection of the aganglionic bowel. However, patients frequently suffer debilitating, lifelong symptoms, with multiple surgical procedures often necessary. Hence, alternative treatment options are crucial. An attractive strategy involves the transplantation of ENS progenitors generated from human pluripotent stem cells (hPSCs).

**Design:**

ENS progenitors were generated from hPSCs using an accelerated protocol and characterised, in detail, through a combination of single-cell RNA sequencing, protein expression analysis and calcium imaging. We tested ENS progenitors’ capacity to integrate and affect functional responses in HSCR colon, after ex vivo transplantation to organotypically cultured patient-derived colonic tissue, using organ bath contractility.

**Results:**

We found that our protocol consistently gives rise to high yields of a cell population exhibiting transcriptional and functional hallmarks of early ENS progenitors. Following transplantation, hPSC-derived ENS progenitors integrate, migrate and form neurons/glia within explanted human HSCR colon samples. Importantly, the transplanted HSCR tissue displayed significantly increased basal contractile activity and increased responses to electrical stimulation compared with control tissue.

**Conclusion:**

Our findings demonstrate, for the first time, the potential of hPSC-derived ENS progenitors to repopulate and increase functional responses in human HSCR patient colonic tissue.

**WHAT IS ALREADY KNOWN ON THIS TOPIC**

- Hirschsprung disease (HSCR) is a devastating condition characterised by aganglionosis of the enteric nervous system (ENS) in the distal bowel, leading to dysmotility, severe constipation and enterocolitis.
- Stem cell therapy offers the potential to generate an ENS in aganglionic tissue and previous studies have described methods for generating ENS progenitors.
- However, the ability of these cells to affect intestinal contractility in HSCR human tissue has not been shown.

**WHAT THIS STUDY ADDS**

- We describe, for the first time, the detailed characterisation of an ENS progenitor population derived from human pluripotent stem cell (hPSC) lines using our efficient protocol.
- Further, we demonstrate the ability of ENS progenitors to differentiate into enteric neurons in vitro and mediate increased functional responses following transplantation into explants of human HSCR tissue.

**HOW THIS STUDY MIGHT AFFECT RESEARCH, PRACTICE OR POLICY**

- These results clearly show the potential of hPSC-derived ENS progenitors in stem cell therapy of HSCR for progression towards clinical trials.
- This study highlights the significant advantages of using human surgical discard tissue for testing the efficacy of stem cell therapies.
- The described ex vivo model can be used to test different therapeutic approaches prior to clinical trials.

## Introduction

The enteric nervous system (ENS) is a complex network of neurons and glia organised into ganglia, which controls numerous vital processes in the gut. Defects in ENS generation during embryonic and fetal development result in various clinical disorders, which are challenging to treat and associated with considerable morbidity and mortality. The most common enteric neuropathy is Hirschsprung disease (HSCR), a devastating congenital disease which affects 1:5000 live births and is characterised by the absence of enteric ganglia in the distal portion of the GI tract.^1 2^ The aganglionic bowel tonically constricts, causing chronic constipation and a swollen ‘megacolon’ proximal to the aganglionic bowel.^3^ Children with HSCR are prone to Hirschsprung disease-associated enterocolitis (HAEC) and bowel perforation,^4^ which can be fatal if untreated. Surgical resection of the aganglionic region, with pull-through of the ganglionic bowel, is currently the only viable treatment option for these children. However, despite surgical intervention, patients often suffer further GI symptoms including HAEC, chronic constipation and faecal soiling among the most common problems,^5 6^ with a significant proportion requiring secondary surgeries to resolve ongoing complications.^4 7^ Moreover, such complex surgery can lead to long-term consequences such as an unexpectedly high frequency of both subfertility and dyspareunia.^8^

HSCR results from a failure in ENS generation. During embryonic/fetal development, the ENS is derived from a transient population of multipotent cells termed the neural crest (NC).^9 10^ These NC cells predominantly migrate from the neural tube at the vagal axial level to acquire ENS progenitor features while colonising the GI tract, giving rise to enteric neurons and glia,^11–13^ with a smaller contribution arising from the sacral NC or via Schwann cell precursors.^14–17^ In HSCR, ENS progenitors fail to migrate, proliferate or differentiate along variable lengths of the distal gut, which remain aganglionic and fail to function.^18^ The underlying mechanisms for this failure remain unclear and are likely multifactorial, although disruption of the RET signalling pathway seems to play a key role in a significant number of cases.^18–21^

A promising strategy for treating HSCR is repopulation of the aganglionic bowel with functional ENS progenitors. Recent proof-of-principle studies have shown unlimited in vitro production of human ENS progenitors from human pluripotent stem cells (hPSCs).^22–25^ In line with this, we have demonstrated the accelerated and efficient generation of vagal NC cells displaying features of early ENS progenitors from hPSCs.^24 25^ These integrated into the mouse GI tract following transplantation and formed ENS-associated neurons and glia.^24^ However, the full therapeutic potential of these hPSC-derived ENS progenitors remains undetermined. Here, we describe the reproducible generation and detailed characterisation of early ENS progenitors from both embryonic and induced pluripotent stem cells (iPSCs). Single cell-RNA sequencing (scRNA-seq) analysis revealed the induction of a population highly expressing vagal NC/early ENS markers, which can be readily directed to differentiate toward functional enteric neurons in vitro. Crucially, we also show that hPSC-derived ENS progenitors can integrate and induce significantly increased contractile activity in patient-derived HSCR colonic tissue following ex vivo transplantation. Collectively, these data suggest that human ENS progenitors produced from hPSCs, via our protocol, are a promising cell population for treating HSCR and other enteric neuropathies.

## Materials and methods

### Stem cell culture and differentiation

Use of hESCs has been approved by the Human Embryonic Stem Cell UK Steering Committee (SCSC15-23). The following hPSC lines were employed: WA09/H9,^26^ H9-RFP,^27^ SFCi55-ZsGr,^28^ MasterShef11^29^ and SOX10:GFP.^30^ All cell lines were cultured routinely in feeder-free conditions in mTeSR1 (Stem Cell Technologies) medium on Geltrex LDEV-Free reduced growth factor basement membrane matrix (Thermo Fisher Scientific). Cells were passaged two times a week after reaching approximately 80% confluency using ReLesR (Stem Cell Technologies) as a passaging reagent. Cells were screened for mycoplasma using Lookout Mycoplasma PCR detection kit (Sigma-Aldrich) or Mycostrip detection kit (Invivogen) and were routinely screened for indicators of pluripotency (OCT4, NANOG, online supplemental table 1) and SSEA4.^31 32^ NC differentiation and enteric neurons were generated as previously described^25^ and detailed in online supplemental methods.

10.1136/gutjnl-2023-331532.supp5 Supplementary data

### Intracellular staining and flow cytometry

Cells were dissociated using TrypLE Select (Gibco), fixed in formaldehyde (PFA, 4% w/v) for 10 min at room temperature (RT) and permeabilised/blocked with blocking buffer containing 0.1% Triton X-100 and bovine serum albumin (1% w/v) in phosphate-buffered saline (PBS) for 1 hour at RT. Primary antibodies were diluted in blocking buffer and cells were incubated with anti-SOX10 (online supplemental table 1) followed by anti-rabbit 488 Alexa fluorophores (A-21206, Invitrogen). Cells were analysed on a FACS Jazz cell sorter (BD). Data were analysed with FlowJo software (BD).

### Single-cell RNA sequencing and analysis

Single-cell RNA sequencing was performed by Single Cell Discoveries (Utrecht, The Netherlands). Cells were differentiated as detailed in online supplemental methods. At days 0, 4 and 6 of differentiation, cells were dissociated using TrypLE Select (Gibco) and cryopreserved in STEM-CELLBANKER (AMSBIO). Library preparation was carried out using the 10× Genomics 3′ V.3.1 kit followed by sequencing on an Illumina Novaseq 6000. Approximately 10 000 cells per time point were analysed at 30 000 reads/cells, 150 bp, paired end. Detailed RNA sequencing analysis can be found in online supplemental methods.

### Calcium imaging

hESC-derived enteric neurons (H9 background) were loaded for 20 min using the Ca^2+^ indicator Fluo-4AM (Invitrogen). Cells were grown on 18 mm glass coverslips and transferred to a specifically designed recording chamber. Images were taken using TILLVISION software (TILL Photonics) with a Zeiss Axiovert 200 M microscope equipped with a monochromator (Poly V) and a cooled CCD camera (Imago QE), both from TILL Photonics. Fluo-4AM was excited at 470 nm and its fluorescence emission collected at 525 nm using a 20× (NA 0.75) objective. Images were collected and analysis was performed using custom written macros in IGOR PRO (Wavemetrics). All recordings were performed at RT. To analyse the recorded images, regions of interest (ROIs) were drawn over each cell and fluorescence intensity was calculated and normalised per ROI to its baseline starting value. Changes in fluorescence intensity were calculated and expressed as a fraction of the baseline fluorescence, as F/F0. For neuron depolarisation, a high K^+^ solution was applied. Cells were also stimulated in an electric field using a 2 s stimulation at 20 Hz (40 stim in total) or on treatment with the nicotinic agonist DMPP (10^−5^ M, Sigma-Aldrich).

### Processing of patient-derived HSCR colonic surgical discard tissue

Human colonic surgical discard tissue from HSCR patients (n=7) was obtained with informed consent through Great Ormond Street Hospital, under local REC approval. Tissue samples were processed under sterile conditions. Briefly, tissue was washed in PBS with Primocin antibiotic (1:500 v/v, InvivoGen) and denuded of the mucosa using fine forceps and microscissors. The serosa was removed along with any fatty tissue/blood vessels. From each patient sample, dependent on sample size, up to a maximum of four tissue segments (approx. 1 cm^2^, harvested from representative points along the oral-anal axis) were isolated for transplantation alongside an equivalent segment (radially adjacent) as a control for sham transplantation. Transplant and sham tissue samples were pinned in sylgard-coated dishes filled with culture media (DMEM F12 with HEPES and glutamine, supplemented with N2 (Invitrogen), B27 (Invitrogen) and Primocin (500 mg/mL, Invivogen)) and placed in a humidified incubator (37°C, 5% CO_2_) overnight.

### Transplantation of ENS progenitors to human HSCR colonic tissue

Frozen vials of ENS progenitors were thawed and resuspended in culture media (approx. 125 000 cells/µL). Cell viability and density was assessed using trypan blue dye (Thermo Fisher Scientific) and an automated cell counter (TC20 Automated Cell Counter, Biorad). Injections into human tissue samples were made using a blunt 5 µL Hamilton syringe attached to a pulled glass capillary needle with a tip diameter of 60 µm. 4 µL cell solution (500 000 cells in total) was injected at a rate of 0.5 µL/min. Following injection, the capillary was kept in place for 1 min before withdrawal. Sham samples received an injection of vehicle-only under the same conditions. Samples were returned to the incubator and maintained for 3 weeks, with media changed every other day.

### Organ bath contractility

Human tissue samples were transferred from culture media to oxygenated Krebs solution and mounted in organ baths (10 mL, SI-MB4; World Precision Instruments). Samples were connected to force transducers (SI-KG20, World Precision Instruments) via sutures (size 4.0, Fine Science Tools) under an initial tension of 1 g. Organ baths were maintained at 37°C and received periodic perfusion of oxygenated Krebs solution. After a 1 hour equilibration period contractile activity was recorded using a Lab-Trax-4 data acquisition system (World Precision Instruments). Tissue samples were subjected to electrical field stimulation (EFS) for 30 s (5 Hz; 40 V; 0.3 ms pulse duration) via platinum electrode loops located at both ends of the tissue sample using a MultiStim System (D330, World Precision Instruments), in the absence or presence of atropine (1 µM, Sigma), L-NAME (100 µM, Tocris Bioscience) and tetrodotoxin (TTX, 1 µM, Tocris Bioscience). Following contractility analysis, samples were harvested for fixation and sectioning.

Contractility data were collected, stored and analysed via Labscribe 2-software (World Precision Instruments) including analysis of baseline contractile responses (frequency and amplitude) and response to electrical stimulation (area under the curve (AUC) for the duration of electrical stimulation). To account for tissue variability all raw responses were normalised to wet tissue weight (g).

### Statistics and data presentation

GraphPad Software (GraphPad Prism) was used to generate all graphs and conduct statistical analysis. Data are expressed as mean±SEM. The significance of functional data was assessed using paired Student’s t-test and one-way analysis of variance (ANOVA) with subsequent intergroup differences determined by Tukey’s multiple comparison tests. P values <0.05 were taken as significant. The individual ex vivo tissue samples examined are reported as ‘segments’. The ‘n values’ reported refer to the number of patients from which tissue samples were harvested. Inter-patient sample pairwise comparisons, between sham-transplanted and ENS progenitor-transplanted human HSCR tissue, were analysed using paired Student’s t-test. To enable analysis across the transplanted cohort (ie, comparison between responses across all patient tissue samples) and to account for variability between patient samples (age range of 2 months to 4.5 years; 6 males and 1 female) functional contractility data were normalised within individual patient groups using feature scaling (min–max):

$$Z=\frac{\left[X-min(X)\right]}{\left[min(X)-max(X)\right]}$$

Normalised results (Z) were then analysed using an unpaired Student’s t-test, with p values <0.05 taken as significant.

Additional methods are provided in online supplemental information.

## Results

### Robust generation of early ENS progenitors from hPSCs

**Figure 1:**
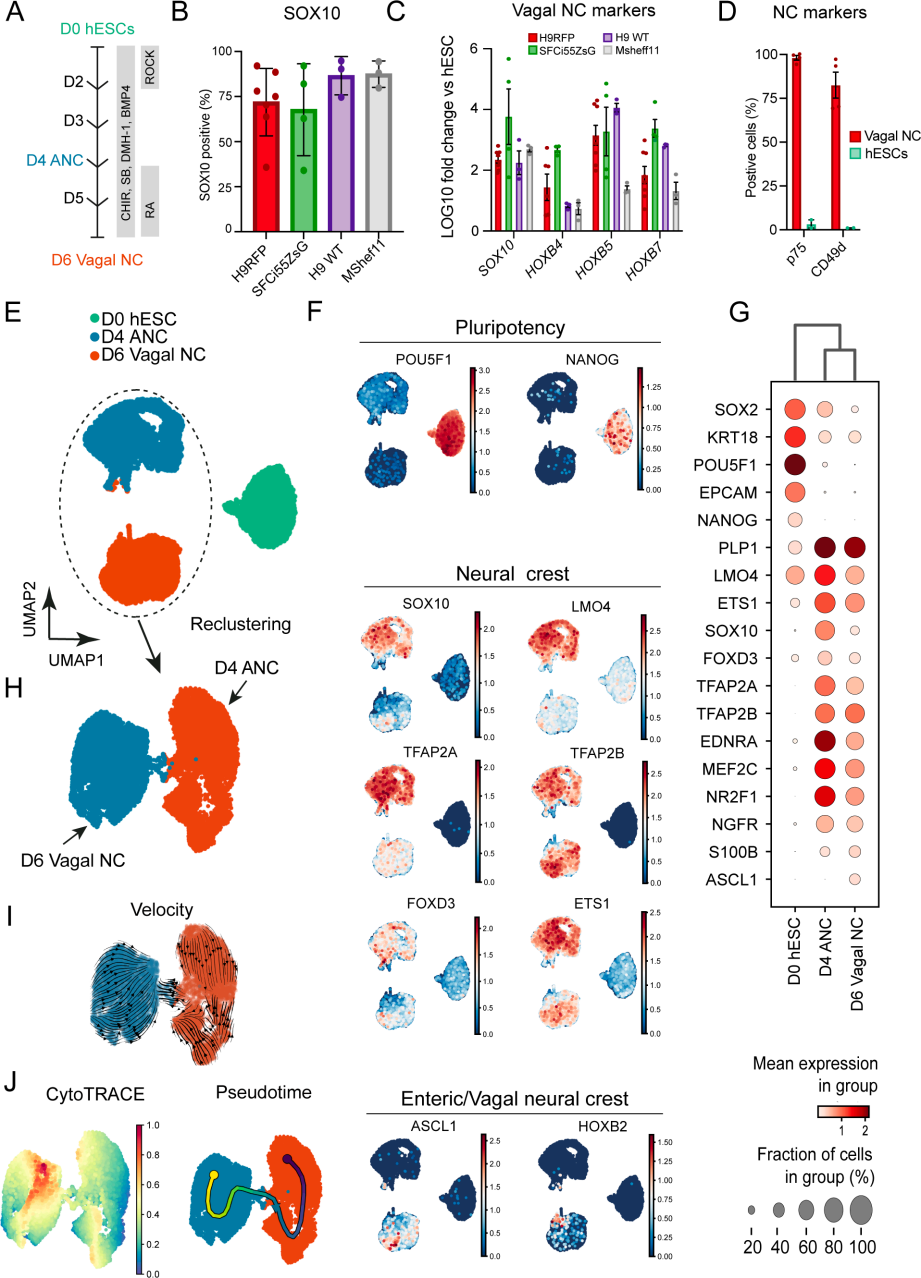
High yields of homogenous vagal NC can be robustly generated from hESCs and induced pluripotent stem cells. (A) Schematic representation of the treatment conditions used to generate vagal NC/enteric nervous system progenitors in vitro. (B) Flow cytometry-based quantification of SOX10 expressing cells in day (D) 6 cultures derived from the indicated human pluripotent stem cell lines. Error bars=SEM (n=3–7 independent differentiations). (C) qPCR expression analysis of *SOX10* and key vagal *HOX* genes carried out in the same D6 cultures as those shown in B (error bars=SEM; n=3–7 independent differentiations). (D) Quantification of p75 and CD49d-positive cells in D6 cultures following immunostaining and flow cytometry (error bars=SEM; n=3). Each biological repeat is represented by a unique symbol. (E) UMAP visualisation of 17 928 cells and their distribution in three samples (D0, D4 and D6) corresponding to unbiasedly defined clusters. (F) UMAP plots showing expression of a set of selected marker genes. (G) Dot plot visualisation of the selected expressed marker genes for each cluster. (H) Selective re-clustering of D4 and D6 samples. (I) RNA velocity analysis of D4 and D6 samples. (J) Cytotrace and pseudotime analysis of D4 and D6 samples. ANC, anterior NC; hESCs, human embryonic stem cells; NC, neural crest.

To evaluate the robustness of our previously published vagal NC/ENS differentiation protocol (figure 1A),^24^ we examined its compatibility with two distinct human embryonic (hESC) lines and one induced pluripotent (iPSC) stem cell line. We employed (i) the female hESC line WA09 (H9), including genetically modified versions containing either a constitutive RFP or GFP reporter under the transcriptional control of SOX10,^27 30^ (ii) the male hESC cell line MasterShef11 (MShef11)^29^ and (iii) the female iPSC line (SFCi55ZsG) marked by constitutive expression of the fluorescent tag ZsGreen, thus enabling cell tracking and visualisation in transplantation experiments.^24 28^ Our protocol initially produces an anterior NC progenitor (ANC) population through combined stimulation of WNT signalling, TGFβ signalling inhibition and moderate BMP activity for 4 days,^33^ followed by addition of retinoic acid (RA) for a further 2 days (figure 1A). Culture of all three cell lines under these conditions efficiently induced the consistent expression of the pan-NC/ENS progenitor marker SOX10^34 35^ by day 6 of differentiation: 60%–80% of all cells were marked by protein expression of SOX10 (figure 1B). qRT-PCR analysis of the resulting cultures further demonstrated that the upregulation of *SOX10* was accompanied by high expression levels of *HOX* paralogous group members indicative of a vagal axial identity (*HOXB4, HOXB5* and *HOXB7*) (figure 1C). Furthermore, flow cytometry analysis of day 6 cultures derived from H9 background hESCs revealed widespread positivity for the NC/ENS progenitors cell surface markers p75 (NGFR) and CD49d (97.9% and 82.3%, respectively; figure 1D). Moreover, we detected minimal presence of ‘contaminating’ central nervous system neurectoderm and non-neural ectoderm cells, marked by the expression of PAX6 and GATA3 proteins, respectively (<1% of total cells; online supplemental figure 1A). Furthermore, we found no evidence of neuronal differentiation, reflected by the absence of PERIPHERIN protein in day 6 cultures (online supplemental figure 1B). Together, these data confirm that our protocol efficiently and rapidly gave rise to high yields of vagal NC/early NC progenitor cells from different hPSC lines.

10.1136/gutjnl-2023-331532.supp1 Supplementary data

We next sought to map the differentiation trajectory of hPSCs toward vagal NC/early ENS progenitors in more detail. To this end, we analysed differentiating hESCs (H9-RFP) at days (D) 0, 4 and 6 by single-cell RNA sequencing (scRNA-seq). We obtained 17 928 cells, which passed quality control (online supplemental figure 1C–G), that were allocated to three distinct clusters corresponding to the analysed differentiation timepoints (figure 1E). The D0 cluster was enriched in pluripotency-associated transcripts (eg, *POU5F1*, *NANOG*), while the vast majority of cells in the D4 cluster exhibited expression of bona fide NC markers such as *SOX10*, *NGFR*, *LMO4*, *ETS1*, *MEF2C* and *TFAP2A/B* (figure 1F,G). Expression of these transcripts, most of which are also present in the developing ENS, was largely maintained in the D6 cluster (figure 1F,G). Cells within the latter uniquely exhibited upregulation of markers specifically denoting a vagal NC/early ENS progenitor state with gliogenic characteristics (*PLP1*, *ASCL1*, *S100β*, *HOXB2*)^36^ (figure 1F,G). Examination of cell differentiation status was achieved by assigning the developmental stage of cells to clusters connected by trajectory using RNA velocity^37^ and also CytoTRACE, a computational framework for predicting differentiation states based on transcriptional diversity.^38^ This revealed a single stable developmental trajectory from D4 ANC cells to D6 vagal NC (figure 1H–J). Collectively, these findings confirm that cultures generated using our protocol are predominantly composed of early ENS progenitors; these appear to emerge exclusively on RA-driven ‘posteriorisation’ of a *HOX*-negative cranial NC state, in line with our previous observations.^39^

**Figure 2:**
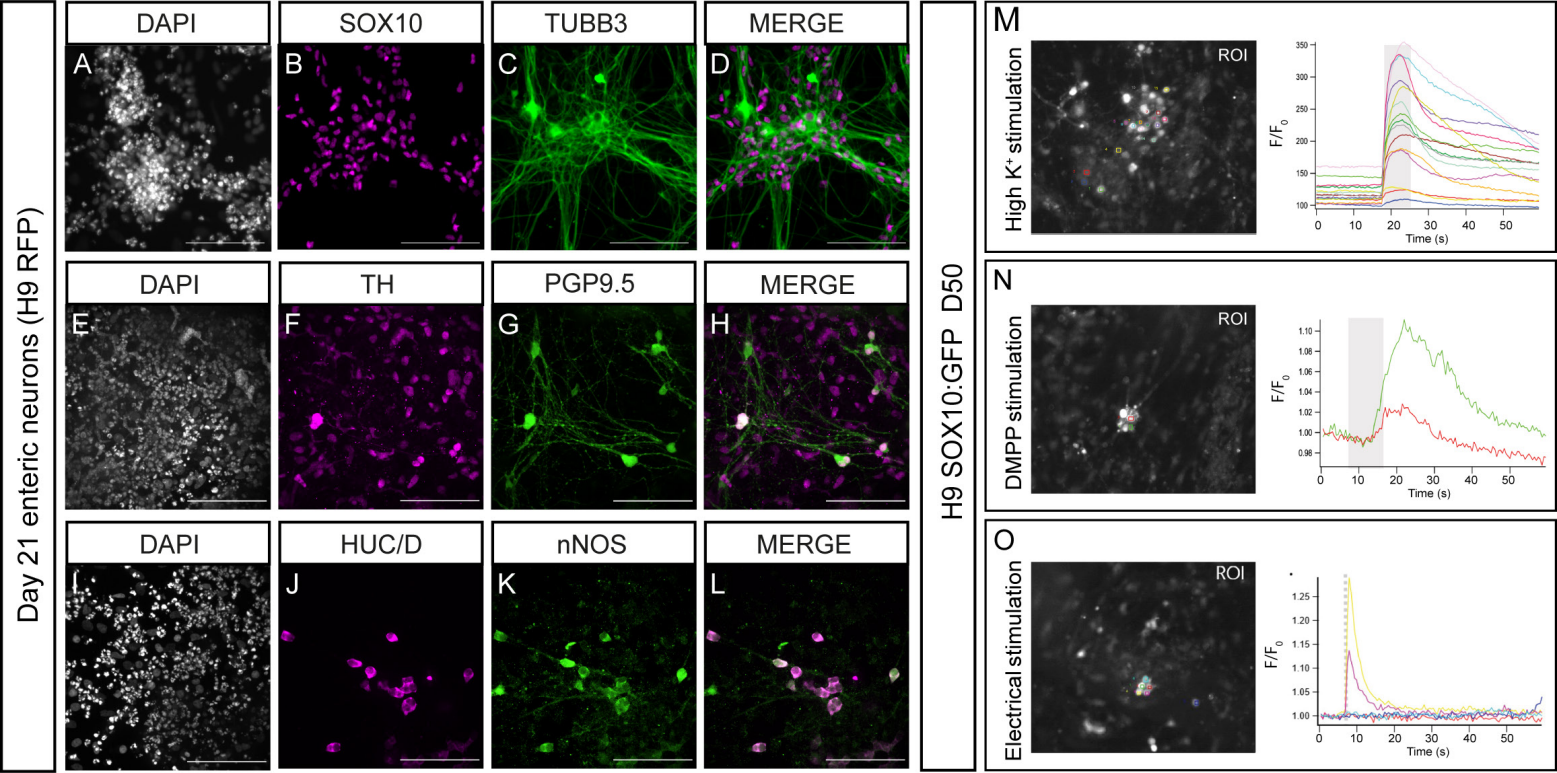
Human embryonic stem cell (hESC)-derived vagal neural crest (NC)/enteric nervous system (ENS) progenitors can be differentiated to enteric-like neurons and glia in vitro. (A–L) Representative images of day 21 neurons immunolabeled with indicated ENS markers following in vitro differentiation of hESC-derived (H9 background) vagal NC cells. (M–O) Analysis and quantification of hESC-derived (H9 background) neuronal Ca^2+^ response to depolarisation with high K^+^ (M), activation with DMPP (N) and electrical stimulation (O). Left: overview of the regions of interest (ROIs) employed for analysis. Right: individual line traces of the responding cells, the change in fluorescence (F/F0) is plotted over time (s). The traces are randomly chosen for illustration. The application of high K^+^/DMPP/electrical stimulation is represented by the grey bar in the graph. Scale bars: 100 µm.

To assess the capacity of our D6 early ENS progenitors (H9-RFP) to generate enteric-like neurons in vitro, we subjected them to ENS differentiation conditions, as previously described.^24 25^ By D21, progenitors gave rise to complex neuronal networks widely expressing ENS progenitor/enteric glial markers (SOX10) and enteric neuronal markers (TUBB3, TH, PGP9.5, HUC/D, nNOS, RET, CALB2 and TRKC) (figure 2A–L, online supplemental figure 2 A,B). Similar results were obtained using the SFCi55ZsG iPSC line (online supplemental figure 2C). Moreover, we found that D6 ENS progenitors could efficiently generate enteric neurons and glia even after cryopreservation and storage in liquid nitrogen for 6 months despite a decrease in viability (online supplemental figure 2D,E). The functionality of in vitro-derived neurons was further examined via calcium imaging. Ca^2+^ transients in ENS progenitor derivatives were observed following application of either high K^+^, treatment with the nicotinic agonist DMPP or EFS (figure 2M–O). Based on these data, we conclude that D6 hPSC-derived vagal NC/early ENS progenitors can be directed to produce in vitro cells that exhibit the hallmarks of functional enteric neurons and glia.

10.1136/gutjnl-2023-331532.supp2 Supplementary data

### hPSC-derived ENS progenitors survive transplantation and migrate through the endogenous tissue

**Figure 3:**
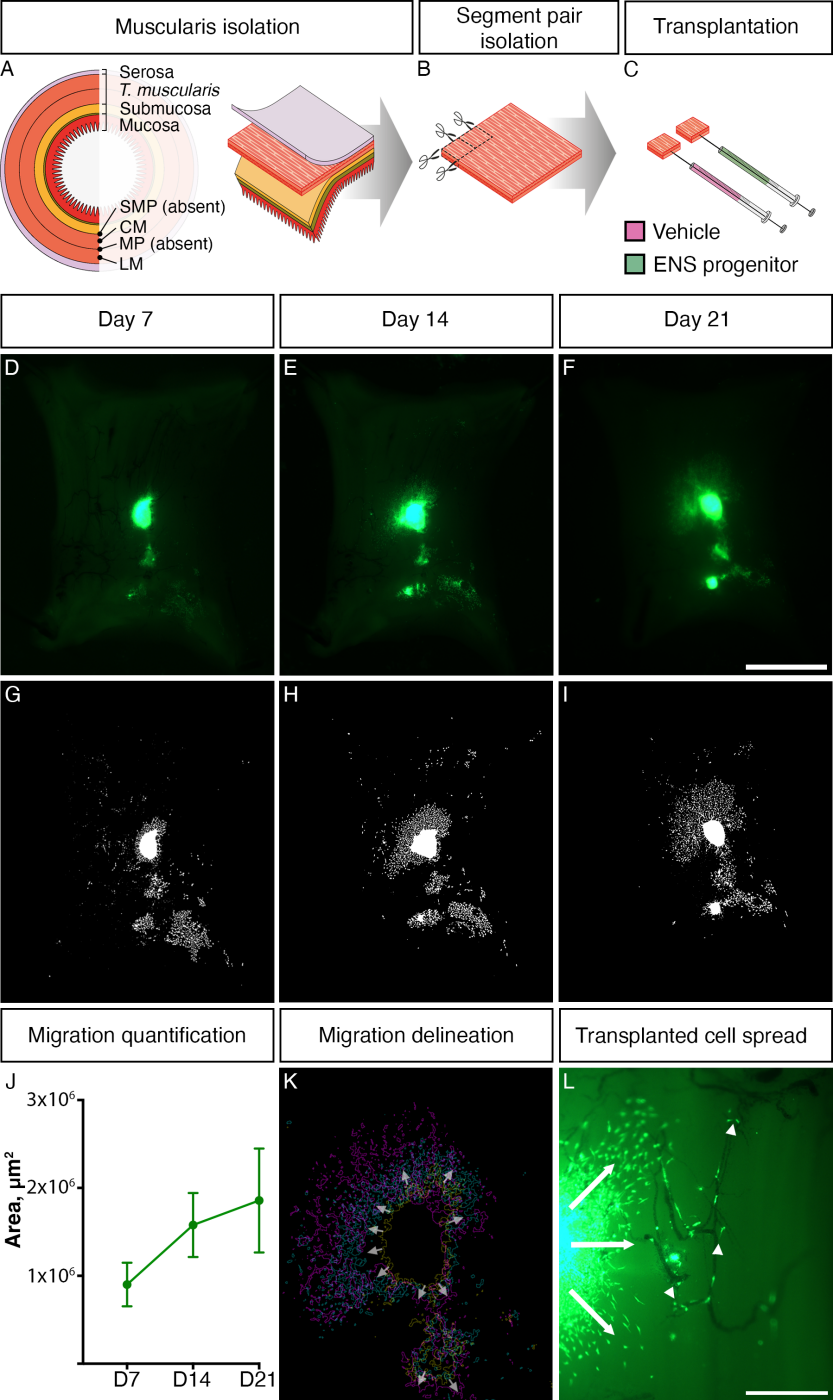
ZsGreen-labelled ENS progenitors survive transplantation into Hirschsprung disease (HSCR) surgical discard tissue and spread through endogenous tissue. (A–C) Schematic representation of ex vivo transplantation procedure conducted on patient-derived HSCR colonic tissue. (D–F) Representative stereoscopic images of ZsGreen^+^ donor ENS progenitor cells following ex vivo transplantation into patient-derived HSCR tissue at (D) day 7 (D7), (E) D14 and (F) D21. (G–I) Representative images showing delineation of ZsGreen^+^ donor cell coverage (from D to F) achieved using a custom-designed FIJI Macro. (J) Summary data showing quantification of donor cell coverage at D7, D14 and D21; n=4 transplanted segments. (K) Representative image showing superimposed outlines of the extent of migration at D7 (yellow outline), D14 (cyan outline) and D21 (magenta outline) from panel D to F. Arrows show direction of spread from the presumptive transplant site. (L) High magnification imaging revealed donor cells migrating from the site of transplantation in all directions (arrows) and ZsGreen^+^ cells that were observed closely associated with endogenous vasculature (arrowheads). Scale bars: D–F: 2 mm; L: 500 µm. CM, circular muscle; ENS, enteric nervous system; LM, longitudinal muscle; MYP, myenteric plexus; SMP, submuscosal plexus.

HSCR patient-derived colonic samples (obtained from surgical discard tissue under informed consent) were transplanted with freshly thawed vials of frozen ENS progenitors generated from either ZsGreen^+^ SFCi55 iPSCs or H9-RFP hESCs and maintained in organotypic culture ex vivo (figure 3A–C). Transplanted segments were imaged at 7-day intervals (ie, D7, figure 3D; D14, figure 3E; D21, figure 3F) throughout ex vivo culture, with ZsGreen expression being maintained and observed in all transplanted segments that were imaged at D21 prior to further processing (15/15). Using a custom-designed FIJI Macro, which unbiasedly delineated the extent of donor ZsGreen^+^ cell integration in recipient tissues, we were able to map donor cell migration following ex vivo transplantation. Importantly, donor cell coverage was observed to increase with time following transplantation: D7 (0.72±0.25×10^6^ µm^2^), D14 (1.26±0.36×10^6^ µm^2^) and D21 (1.49±0.59×10^6^ µm^2^) (figure 3J, n=4). Moreover, delineation and superimposition of the maximal perimeter of donor cell coverage at D7, D14 and D21 revealed progressive migration in all directions from the presumptive site of transplantation (figure 3K). Interestingly, donor ENS progenitors appeared to migrate, in all directions from the transplant site, as individual cells or in ‘chains’ reminiscent of NC cells/ENS progenitors colonising the embryonic murine GI tract in vivo.^40^ These migrating transplanted cells were often detected in close association with the presumptive endogenous tissue vasculature (figure 3L, arrowheads).

### hPSC-derived ENS progenitors integrate and form neurons in human HSCR colon following ex vivo transplantation

**Figure 4:**
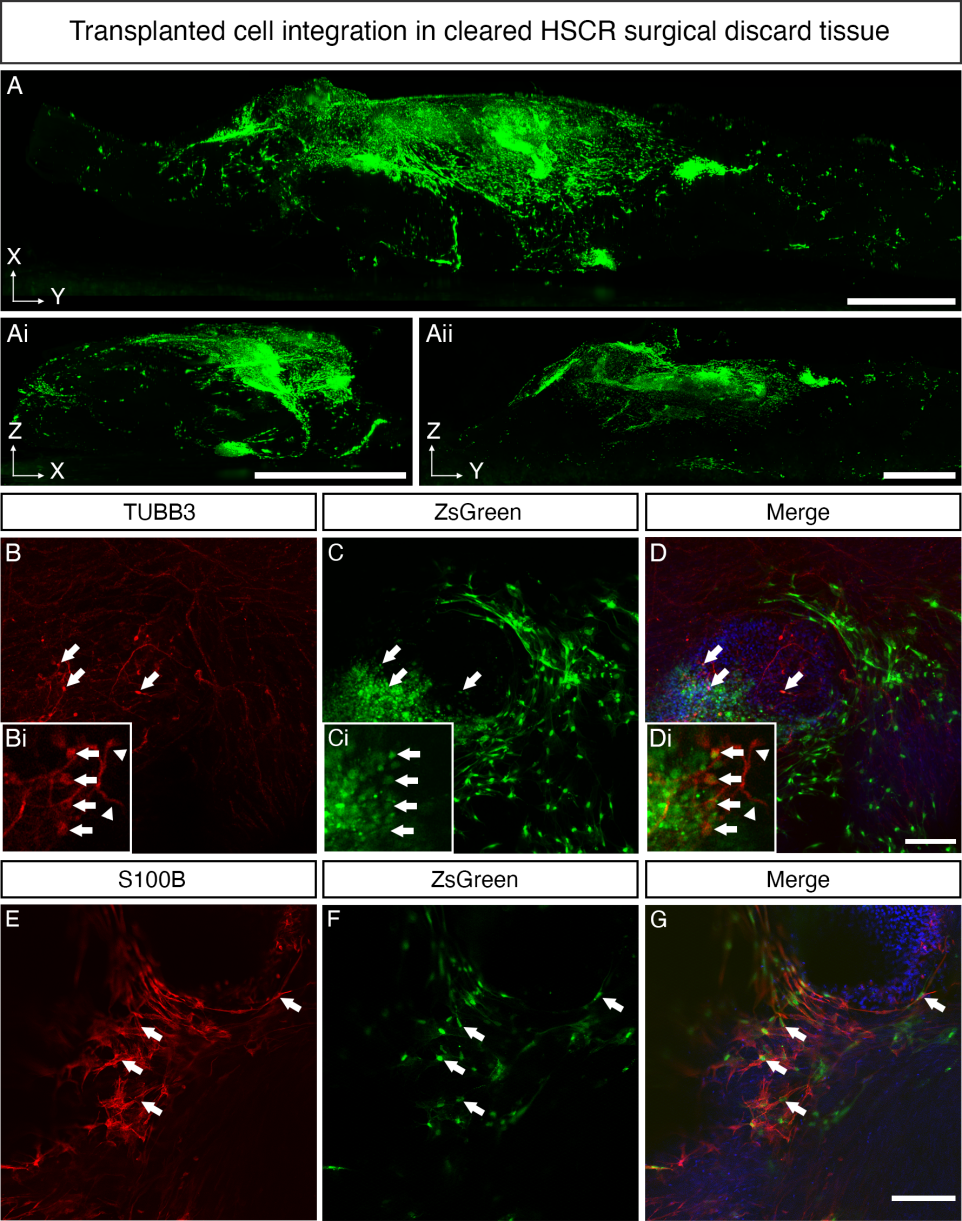
Enteric nervous system (ENS) progenitors transplanted into Hirschsprung disease (HSCR) surgical discard tissue differentiate into neurons and glia. (A–Aii) Representative images, acquired by light sheet fluorescence microscopy, of cleared HSCR patient-derived tissue at D21 post-transplantation in three planes: XY (A), ZX (Ai) and ZY (Aii). ZsGreen^+^ ENS progenitors could be seen clustered around the initial transplant site and migrating from the transplant site in streams. (B–D) Representative confocal images of cleared transplanted tissue at D21 demonstrating the presence of ZsGreen^+^TUBB3^+^ cells within recipient HSCR patient-derived tissue segments (arrows). Insets show TUBB3^+^ transplanted cells captured at higher magnification, extending axon-like processes (arrowheads). (E–G) ZsGreen^+^S100β^+^ donor-derived cells were also readily detected. Scale bars: A–Aii: 1 mm; D, G: 100 µm.

To assess donor cell differentiation, transplanted tissue segments were cleared using FluoClearBABB^41^ (figure 4). Light sheet fluorescence microscopy analysis of D21 transplanted tissues revealed substantial ZsGreen^+^ donor cell integration within the cleared recipient tissue in all three dimensions (XY: figure 4A; ZX: figure 4Ai; YX: figure 4Aii, online supplemental figure 3) suggesting migration of donor-derived cells both along and into the recipient tissue following transplantation. We next determined whether ENS progenitors transplanted into the HSCR colonic tissue microenvironment retained the neuroglial potential demonstrated in vitro. To achieve this, immunohistochemistry was performed for the neuronal marker TUBB3 and glial marker S100β. At D21, ZsGreen^+^TUBB3^+^ donor cells were readily detected within HSCR colonic tissue (figure 4B–D, arrows) with differentiated donor cells displaying neuronal morphology, including the extension of TUBB3^+^ axonal processes which formed branching interconnections reminiscent of rudimentary neuronal circuits (figure 4Bi–Di, arrowheads). ZsGreen^+^S100β ^+^ donor cells were also readily detected (figure 4E–G, arrows). We also sought to examine the transcriptional identity of donor derived cells following transplantation. To do this, ZsGreen^+^ donor cells were isolated via FACS, pooled and the expression of target transcripts assessed using qPCR/RT-PCR (online supplemental figure 4). Cells harvested at D21 post-ex vivo transplantation displayed a 7.99-fold and 1.78-fold increase in the expression of *TUBB3* and *S100β*, respectively when compared with D6 ENS progenitors (ie, the starting donor cell population used at transplantation). By contrast, expression of *SOX10* in isolated donor derived cells at D21 was reduced to 0.041 compared with normalised expression in D6 ENS progenitors (online supplemental figure 4B). The expression of both *CALB2* and *NOS1* was also detected in isolated donor-derived cells post-transplantation, suggesting the emergence of enteric neuronal subtypes (online supplemental figure 4C).

10.1136/gutjnl-2023-331532.supp3 Supplementary data

10.1136/gutjnl-2023-331532.supp4 Supplementary data

Collectively, these results indicate that hPSC-derived ENS progenitors can efficiently migrate, integrate and differentiate toward ENS cell types within human HSCR colonic tissue ex vivo.

### Transplantation of hPSC-derived ENS progenitors increases functional responses in HSCR colonic tissue

**Figure 5:**
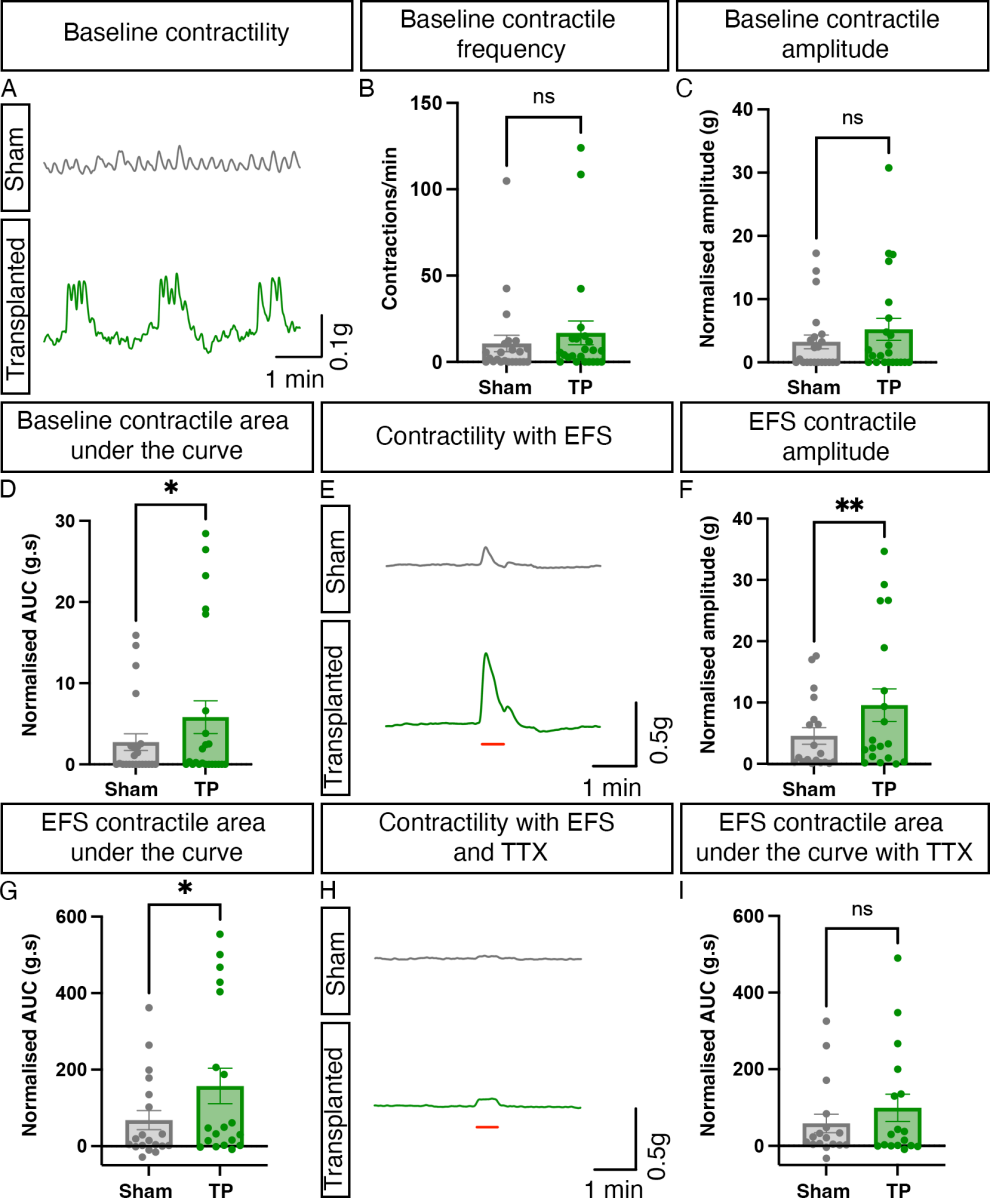
Transplantation of enteric nervous system (ENS) progenitors into Hirschsprung disease (HSCR) surgical discard tissue increases both baseline contractility and response to electrical stimulation via neuronal activity. (A) Representative traces showing basal contractile activity in HSCR patient-derived tissue 21 days after sham (grey) or ENS progenitor-transplantation (green). (B–D) Summary data of baseline contractile frequency (B), amplitude (C) and cumulative area under the curve (AUC; D). (E) Representative contractility traces of sham (grey) or ENS progenitor transplanted (green) tissues in response to electrical field stimulation (EFS; red bar) in control conditions. (F, G) Summary data of amplitude (F) and AUC (G) in response to EFS in control conditions. (H) Representative contractility traces of sham (grey) or ENS progenitor-transplanted (green) tissues in response to electrical field stimulation (EFS; red bar) in the presence of 1 µM tetrodotoxin (TTX). (I) Summary data of AUC in response to EFS in TTX. To account for variability in tissue size, all functional analyses (peak contractile amplitude (g) and area under the curve (g.s) measurements) were normalised to wet tissue weight (g). *p<0.05. Error bars=SEM.

The inability of aganglionic colonic tissue to contract and relax in a controlled manner is a key feature of HSCR. As transplanted ENS progenitors were found to integrate and differentiate within the explanted HSCR colon, we next examined their ability to alter the functional responses of the host tissue using organ bath contractility. Prior to assessment, ZsGreen expression was visually confirmed in all transplanted segments and no samples were excluded from initial functional assessment. However, three paired sections (ie, one sham segment and the equivalent transplanted segment from three individual patients) were excluded from subsequent analysis due to technical issues in experimental preparations. Organ bath contractility, performed on transplanted HSCR colonic segments and spatially adjacent sham-injected controls, revealed significant improvements in baseline contractility and response to EFS (figure 5A–G). Basal contractile frequency was comparable between ENS progenitor-transplanted segments and sham-transplanted control tissue (10.7 and 16.9 contractions/min, respectively, p=0.2305, figure 5B, n=7 patients, pairwise comparison of 21 ENS progenitor-transplanted and sham-transplanted segments). Similarly, we detected no difference in normalised basal contractile amplitude between ENS progenitor-transplanted segments and sham-transplanted control tissue (3.2 and 5.2 g, respectively, p=0.0887; figure 5C, n=7 patients, pairwise comparison of 21 paired segments). However, complex basal motor contractions were observed more frequently in hPSC-derived ENS progenitor-transplanted tissue compared with sham-transplanted tissue (61.9% and 42.9%, respectively, n=7 patients, 21 paired segments). This resulted in a significant increase in the cumulative magnitude of basal contractions in ENS progenitor-transplanted tissue compared with sham-transplanted tissue (normalised AUC: 5.8 and 2.7 g.s, respectively, p=0.015; figure 5D, n=7 patients, 21 paired segments). Additionally, ENS progenitor-transplanted tissue elicited a significantly increased contractile response to EFS when compared with sham-transplanted controls (figure 5E–G). Normalised contractile amplitude was significantly increased in ENS progenitor-transplanted tissue compared with sham-transplanted controls (9.6 and 4.6 g, respectively, p=0.0062; figure 5F, n=7 patients, 19 paired segments). Similarly, we observed a significant increase in contractile magnitude in ENS progenitor-transplanted tissue compared with sham-transplanted controls (normalised AUC: 157.4 and 68.0 g.s, respectively, p=0.0141; figure 5G, n=7 patients, 19 paired segments). Notably, following application of TTX (1 µM), a potent neurotoxic sodium channel blocker, ENS progenitor-transplanted tissue and sham-transplanted tissue displayed comparable responses to EFS (normalised AUC: 99.5 and 58.6 g.s, respectively, p=0.0887; figure 5H,I, n=7 patients, 17 paired segments).

**Figure 6:**
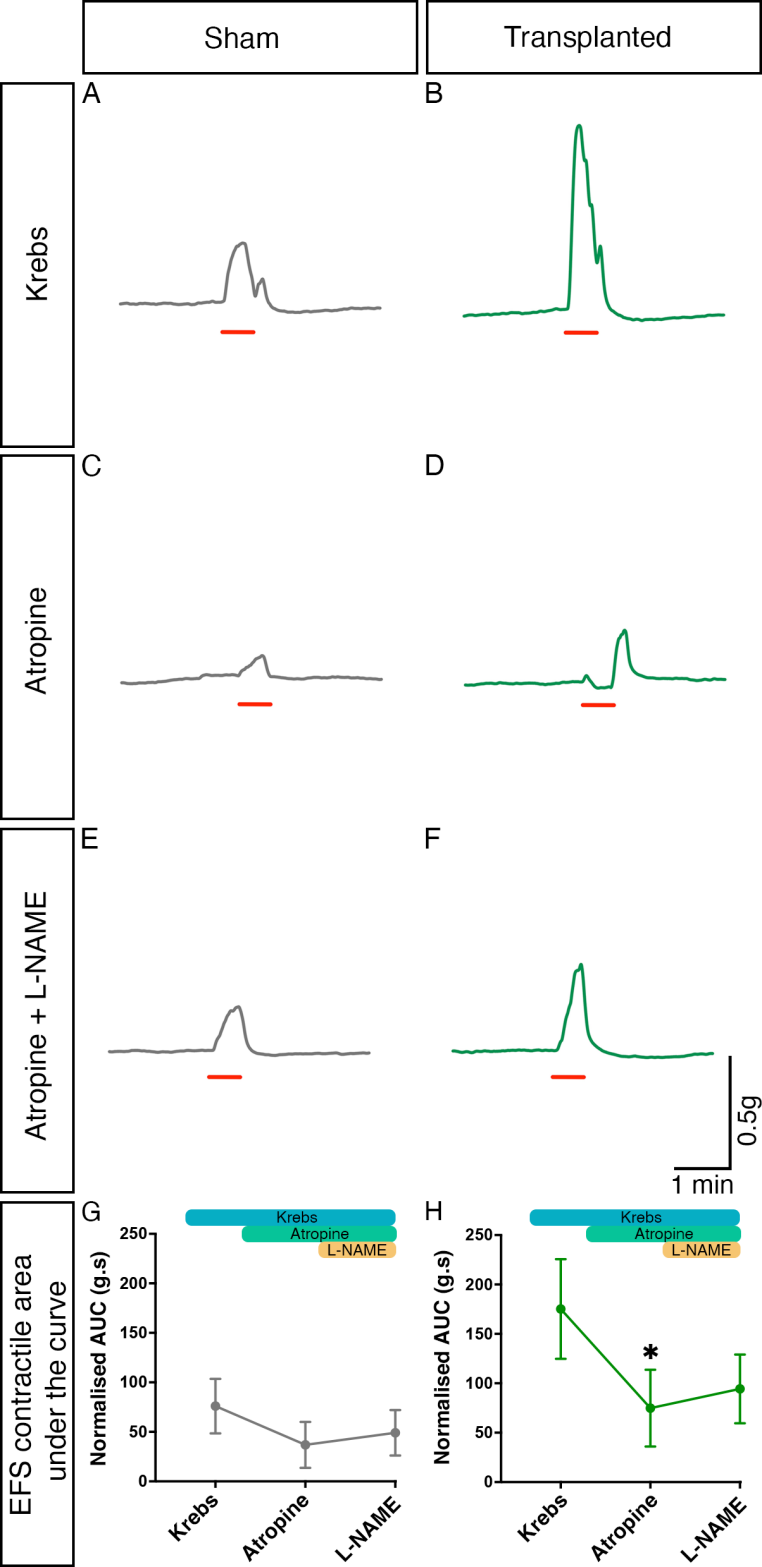
Cholinergic and nitrergic activity contribute to contractile responses within transplanted Hirschsprung disease tissue. (A–F) Representative contractility traces of sham (grey) or enteric nervous system (ENS) progenitor transplanted (green) tissues in response to electrical field stimulation (EFS; red bar) in control conditions (A, B), in the presence of atropine alone (C, D) and in the presence of atropine+L NAME (E, F). (G, H) Summary analysis of normalised area under the curve (AUC) response in sham (G) or ENS progenitor-transplanted (H) tissues. To account for variability in tissue size, area under the curve (g.s) measurements were normalised to wet tissue weight (g). *p<0.05. Error bars=SEM.

We also sought to identify the roles of both cholinergic and nitrergic innervation in the observed increased response to EFS via sequential pharmacological blockade with atropine and L-NAME. Both atropine and L-NAME were found to affect contractile responses to EFS in both sham and transplanted segments (figure 6A–F). Importantly, one-way ANOVA suggested that the responses observed in sham tissue in the presence of atropine, and subsequently L-NAME, were comparable to those in control conditions (F(1.08, 17.25)=1.510, p=0.2380; n=7 patients, 17 sham segments; figure 6G). By contrast, within transplanted segments significant changes in responses were observed with pharmacological application by one-way ANOVA (F(1.50, 24.00)=5.693, p=0.0151; n=7 patients, 17 transplanted segments, figure 6H). Post-hoc Tukey’s comparison revealed a significant difference between the mean response in control conditions (normalised AUC: 175.2 g.s, n=7 patients, 17 transplanted segments) compared with that observed in the presence of atropine (74.83 g.s, n=7 patients, 17 transplanted segments, p=0.0406). Subsequent application of L-NAME, in the presence of atropine, led to an increased mean contractile response (94.31 g.s, n=7 patients, 17 transplanted segments) though this was not statistically different from the responses observed in either control conditions (p=0.0731) or following the application of atropine alone (p=0.6284).

**Figure 7:**
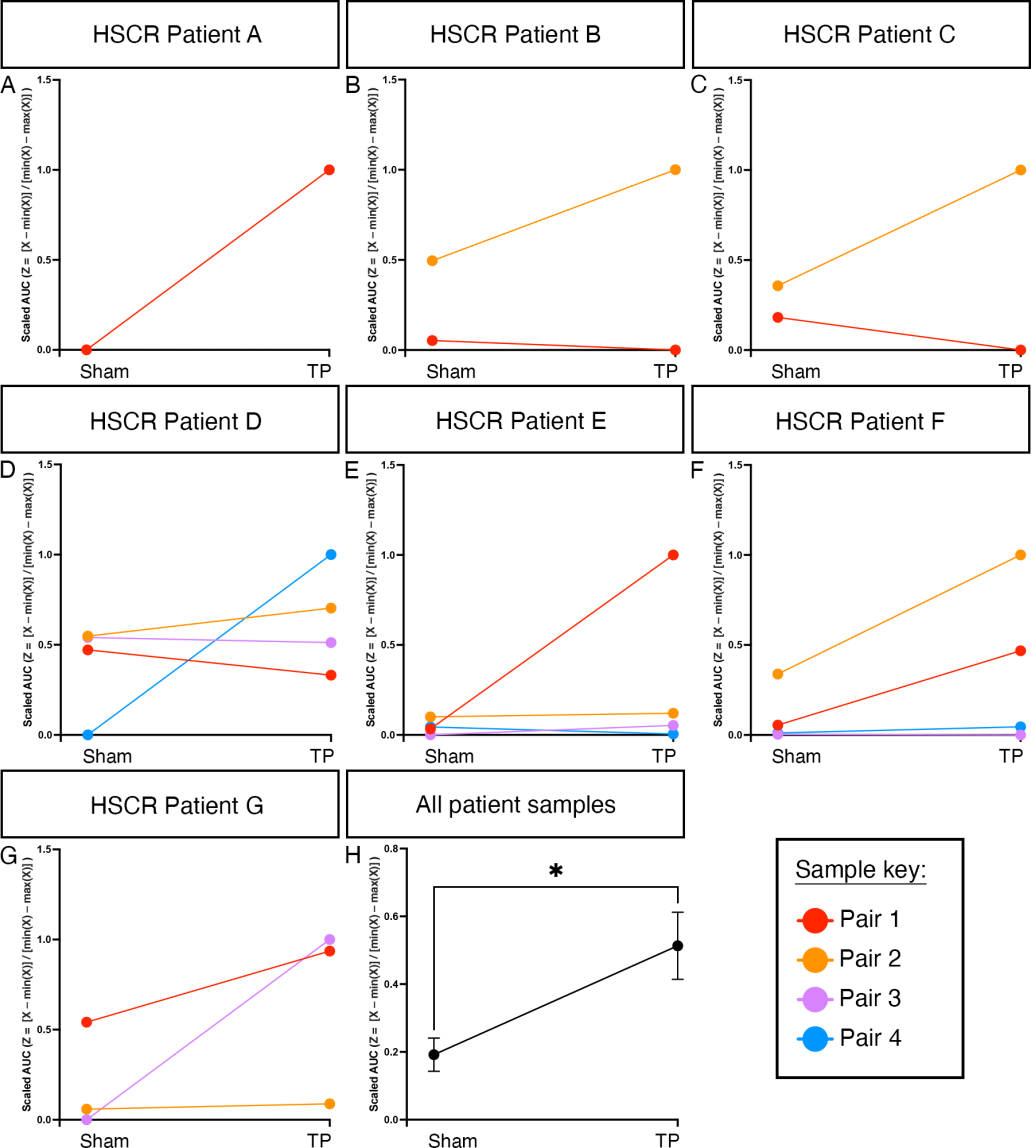
Enteric nervous system (ENS) progenitor transplantation increases the response of Hirschsprung disease (HSCR) surgical discard tissue samples to electrical stimulation. To account for patient variability (in terms of age, gender, disease severity and positioning of the sample along the oro-anal axis) samples were normalised by patient using feature scaling (Z=[X–min(X)]/[min(X)–max(X)]). (A–G) Slope analysis for each pair of tissue samples under examination by patient. (H) Summary data showing scaled area under the curve (AUC) analysis of ENS progenitor-transplanted samples compared with sham-transplanted. *p<0.05. Error bars=SEM.

We next sought to determine if the observed improvement between paired tissue samples during EFS could be extrapolated at an inter-patient level, taking into account variability in patient age, gender, disease severity and resected tissue size. To achieve this, we employed feature scaling whereby functional responses were normalised across each patient. This revealed a positive increase in the response to EFS in 68.4% of ENS progenitor-transplanted HSCR segments compared with sham-transplanted controls (figure 7A–G, n=7 patients, 19 paired segments). Promisingly, using this analysis, an overall improvement in the response of ENS progenitor-transplanted segments compared with sham-transplanted controls could be detected at a group level (average slope gradient=+0.3573) which was found to be significant when compared with zero (ie, no effect, p=0.0108; figure 7H, n=7 patients). These data suggest an average positive improvement in contractile response to EFS across the entire patient cohort in our study. Collectively, our findings indicate that transplantation of hPSC-derived ENS progenitors triggers neuronally mediated functional responses in HSCR patient-derived colonic tissue.

## Discussion

HSCR is a debilitating condition with no cure. Although surgery is lifesaving, many HSCR patients suffer persisting symptoms even following surgical intervention.^42^ Recent advances in stem cell therapy^43 44^ offer the potential to replenish missing neurons following surgery and bridge connections in the newly anastomosed colon to mitigate these ongoing symptoms and possibly, one day, to even remove the need for surgical resection entirely. Previous data have highlighted the potential of allogenic^45 46^ and isogenic hPSC-derived ENS progenitors^22 23^ to integrate and rescue murine models of colonic dysfunction. Here, we demonstrate that transplantation of ENS progenitors, generated from hPSCs using our previously described protocol^24 25^ and subsequent differentiation in situ, as evidenced by detection of donor-derived neurons, can significantly increase functional contractility in human HSCR surgical discard tissue. To our knowledge, the results described here represent the first improvement-of-function of human HSCR tissue. We show an increase in the magnitude of basal contractility as well as increased responses to electrical stimulation following ENS progenitor transplantation. Crucially, this finding addresses a key outcome for evaluation of stem cell transplantation efficacy in HSCR, in line with the accepted standards laid out in a ‘white paper’.^47^

We show that our protocol can reproducibly and efficiently give rise to high yields of ENS progenitors from multiple hPSC lines within 6 days. This is quicker than previously published protocols that yield ENS progenitors after 10–15 days at a comparable efficiency^22 48^ thus reducing potential manufacturing costs, a key advantage for the development of a viable cell therapy against HSCR. Our data also indicate that the resulting ENS progenitors can be cryopreserved and recovered with high efficiency which is essential for multicentre clinical application of this technology. Importantly, following recovery, cells maintained their ability to differentiate to ENS lineages and could be used for ex vivo cell transplantation into HSCR colonic tissue. This is a crucial feature for the development of an ‘off-the-shelf’ therapy of hPSC-derived ENS progenitors which overcomes a key technical barrier to clinical translation. A relatively modest number of transplanted cells (500 000/cm^2^) was sufficient to promote positive functional change, in line with previous studies showing that small numbers of transplanted cells can exert a dramatic functional effect.^49 50^ We also determined that this functional improvement was neuronally mediated, as demonstrated by the inhibitory effect of TTX on the contractile response of ENS progenitor-transplanted segments. Further, we showed that the cholinergic antagonist atropine and the nitrergic antagonist L-NAME also affected contractile responses in ENS progenitor-transplanted segments, suggesting the presence of these neuronal subtypes in ENS-progenitor transplanted tissue. Importantly, isolation and analysis of donor-derived cell populations 21 days following transplantation suggested a shift in the molecular identity of retrieved donor cells when compared with our initially transplanted ENS progenitor population: including the upregulation of *TUBB3* and *S100β* and concomitant downregulation of *SOX10* alongside expression of key neuronal subtype transcripts. Interestingly, responses to EFS were also observed in sham-transplanted HSCR segments. These tissues likely include hypertrophic extrinsic nerve bundles on resection, or may include a small component of transition zone, which would account for the observed responses. Nevertheless, our data indicate that the observed increase in contractile behaviour in transplanted HSCR segments is likely to be a result of de novo neurogenesis, driven by transplanted ENS progenitors. This conclusion is supported by previous observations detailing autologous ENS-derived cell integration and neuronal differentiation in HSCR patient tissue ex vivo.^51^ While the addition of TTX led to a significant reduction in contractile responses to EFS in transplanted segments, the degree of this reduction was theoretically smaller than expected. We hypothesise that this finding may be due to the presence of TTX-insensitive donor-derived neurons which have not yet fully differentiated to their mature form, in our ex vivo model system.^52^ However, the possibility of donor cell-mediated non-cell autonomous effects cannot be excluded.^45^ Our protocol gives rise to cultures that are predominantly (67%–87% of total cells) composed of SOX10-positive vagal NC/early ENS progenitors. scRNA-seq analysis of the remaining SOX10-negative fraction indicates that it likely represents more differentiated posterior cranial/vagal NC derivatives and in the future, it will be important to test the effect of the remaining SOX10-negative fraction on the capacity of the transplanted cells to induce functional rescue. Moreover, a recent study has indicated that combined grafts of hPSC-derived vagal and sacral NC cells may be the optimal route toward the development of a cell therapy against HSCR^23^ and therefore it would be important to test in the future the effect of mixing our vagal NC/early ENS progenitors with hPSC-derived sacral NC cells.

We describe the use of human HSCR patient-derived organotypic tissue. This circumvents some of the issues encountered with HSCR mouse models (eg, intramodel and intermodel variability in disease severity and short life span), which have slowed efforts to date. Crucially, our organotypic transplantation approach offers a number of advantages which may be useful for future studies including: (i) the ability to temporally track transplanted cell integration, (ii) the potential to alter cell culture dynamics to affect cell integration/differentiation, (iii) an overall reduction in the use of animal-based assays and most importantly (iv) the observations achieved through this approach are directly translatable. Remarkably, we were able to achieve extended ex vivo human intestinal tissue survival with functional readouts from patient-derived colonic tissue after 3 weeks in culture, providing a powerful preclinical tool for the optimisation of innovative therapies prior to in vivo transplantation. However, this model has some technical limitations. While the approach provides a test bed to examine ENS progenitor integration and effects in patient-derived tissue, the relatively small segments (1 cm^2^), used herein, limit understanding of how donor cells may behave in larger specimens. For example, the significant improvements in contractility described here need to be further examined to determine the development of appropriate donor circuitry and the interactions with native circuitry (both intrinsic and extrinsic) which are required to drive complex motile behaviour in a healthy colon. Additionally, to maintain culture sterility for extended periods it was necessary to remove the mucosal layer prior to transplantation which may impact microenvironmental factors and thus donor cell behaviour. Importantly, given the ex vivo nature of the model system, signalling components and responses (eg, immune cells and inflammatory responses) which may influence donor cell efficacy are excluded from our observations. It will be crucial to conduct further studies into donor cell interactions along with microenvironmental and immunological effects on ENS progenitors before any ENS cell therapy can be progressed to the clinic.

In conclusion, our findings strongly suggest that hPSC-derived ENS progenitors can serve as the basis for the further development of cell therapies aiming to treat conditions characterised by neuronal loss or dysfunction in the gastrointestinal tract, such as HSCR.

## Data Availability

Data are available upon reasonable request. The data that support the findings of this study are available from the corresponding authors upon reasonable request. Transcript profiling: RNA-seq data has been deposited in the Gene Expression Omnibus database (https://www.ncbi.nlm.nih.gov/geo/) under the accession number GSE252061.
