# Supplementary material for: Human enteric nervous system progenitor transplantation improves functional responses in Hirschsprung disease patient-derived tissue
Source: Gut. 2024 May 30;73(9):1441–53. doi: 10.1136/gutjnl-2023-331532 (PMC11347211; doi:10.1136/gutjnl-2023-331532)
Supplement: Supplementary data [file gutjnl-2023-331532supp005.pdf]

Human enteric nervous system progenitor transplantation improves functional responses in Hirschsprung Disease patient-derived tissue

SUPPLEMENTARY FIGURE LEGENDS

Figure S1: Quality control of single cell RNA sequencing

(A) Quantification and representative images of PAX6 and GATA3-positive cells in day (D) 6 neural crest and positive control samples (hPSC-derived neuroectoderm (NE) for PAX6 and sympathoadrenal progenitors (SAP) for GATA3) following immunostaining and image analysis. Error Bars = SEM (n=6). Scale bar = 100μm. (B) Representative images of Peripherin in D6 ENS progenitors and in positive controls (Day 21 ENS neurons). (C) Plotted number of detected UMIs, genes and percentage of mitochondrial genes per cell before and after filtration. (D) Scatterplot of cells based on the first two principal components and contribution of single PCs to the total variance in the data. (E-G) Application of Scrublet software to D0, D4 and D6 datasets for removing the cell doublets. The left column shows histograms of score distribution for observed cells, the right column shows histograms of score distribution for simulated doublets.

Figure S2: hPSC-derived vagal NC/ENS progenitors can be differentiated to enteric-like neurons and glia *in vitro*

(A) Representative images of Day (D) 21 enteric neurons immunolabeled with indicated ENS markers following *in vitro* differentiation of H9-RFP. Scale bar = 100μm. (B) qRT-PCR analysis of D21 ENS neuronal markers. Error bars = SEM; n=3 independent differentiations. (C) Representative images of D21 enteric neurons immunolabeled with indicated ENS markers following *in vitro* differentiation of SFCi55 iPSCs. Scale bar = 100 μm. (D) Viability of ENS progenitors pre- and post- cryopreservation. (E) Representative images of D21 enteric neurons following cryopreservation and storage for 6 months. Neurons were immunolabeled with an indicated neuronal (TUBB3) and glial (S100β) marker following *in vitro* differentiation.

Figure S3: 3-dimensional reconstruction of donor cell integration within Hirschsprung disease patient derived tissue

Representative 3-dimensional reconstruction of ZsGreen$^+$ donor cell integration at D21 obtained using LSFM. Scale bar 1mm.

Figure S4: Donor-derived cells express key enteric neuronal subtype specific transcripts following transplantation into HSCR tissue

(A) Representative FACS plot showing gating used to isolate ZsGreen$^+$ at D21 post transplantation. (B) Summary data showing 2^DCt calculation of gene expression in ZsGreen$^+$ donor-derived cells at D21 post transplantation compared to D6 ENS progenitors. Dashed line represents normalised gene expression levels in D6 ENS progenitors. (C) Representative image of PCR analysis of *GAPDH*, calretinin (*CALB2)* and neuronal nitric oxide synthase (*NOS1*) in isolated ZsGreen$^+$ donor derived cells at D21 post transplantation.

SUPPLEMENTARY METHODS

Patient and Public Involvement

Elements of this work have been presented to the Young Person's Advisory Group (YPAG) based at Great Ormond Street Hospital, London, UK as part of an ongoing process to establish a focus group of people affected by Hirschprung Disease.

Stem cell culture and differentiation

Pluripotent stem cells were plated on Geltrex LDEV-Free reduced growth factor basement membrane matrix (Thermo Fisher) or Vitronectin (Thermo Fisher) at a density of 40,000 cells/cm^2 in neural crest differentiation basal medium containing Dulbecco′s Modified Eagle′s Medium (DMEM) F12 (Merck Life Science), and 1x N2 supplement (Gibco), 1x B27 (Gibco),

1x GlutaMAX (Gibco), 1x Minimum Essential Medium Non-Essential Amino Acids (MEM NEAA) (Gibco). The neural crest differentiation basal medium was supplemented with CHIR (1μM, Tocris), SB431542 (2μM, Tocris), DMH-1 (1μM, Tocris), Human BMP4 Recombinant Protein (20ng/ml Thermo Fisher) and Rho-associated coil kinase (ROCK) inhibitor Y-27632 2HCl (10μM, Adooq Biosciences). Supplemented neural crest differentiation medium (CHIR, DMH-1, SB431542 and BMP) was replaced on days 2 and 3 without ROCK inhibitor. On days 4 and 5, media was replaced with supplemented neural crest medium (CHIR, DMH-1, SB431542 and BMP) plus retinoic acid (RA, 1μM, Merck). For cryopreservation, cells were resuspended in 0.5 ml STEMCELL-BANKER (AMSBIO) and incubated at -80°C for 24 hours before transfer to liquid nitrogen. To recover for *in vitro* ENS differentiaton, cells were washed in Dulbecco′s Modified Eagle′s Medium (DMEM)/F12 (Merck Life Science). Viability was measured using a trypan blue exclusion assay.

For ENS differentiation, day 6 ENS progenitors were dissociated into single cells using TrypLE Select (Gibco) and replated into an Ultra-Low Attachment 6 well plate (Corning 3471) in media containing a 1:1 mix of DMEM/F12 (Sigma) and Neurobasal media (ThermoFisher 21103049), supplemented with 1x N2 (Gibco), 1x B27 (Gibco), 1x NEAA (Gibco), 1x Glutamax (Gibco), 3μM CHIR99021 (Tocris), 10ng/ml FGF2 (R&D systems 233-FB/CF), 10μM Y-27632 dihydrochloride (Tocris) and 1μM RA. Cells were subjected to a half medium change 48h following sphere plating and spheres formed by 72h. At 72h, spheres were plated onto Geltrex-coated plates in BrainPhys media (Stem Cell Technologies, 05790) supplemented with 1x N2, 1x B27 (ThermoFisher, 17504044), 100μM Ascorbic Acid (Sigma, A8960), 10ng/ml GDNF (Peprotech, 450-10) and 10μM DAPT (Sigma, D5942). Media was changed every other day until day 21.

Immunofluorescence and imaging of differentiating cell cultures

Cells were fixed in formaldehyde (PFA, 4% w/v) for 10 min at RT and permeabilised/blocked with blocking buffer for 1-2hr at RT. Primary antibodies were diluted in blocking buffer and cells were incubated with primary antibodies overnight at 4°C. Following PBS washes, cells were incubated with secondary antibodies diluted in blocking buffer (Invitrogen). Cell nuclei were counterstained with DAPI:PBS (Thermo Fisher, 1:12,000) and fluorescent images were acquired using the InCell Analyser 2200 system (GE Healthcare) or a Nikon W1 spinning disk confocal micrscope. Images were processed in Fiji (1). Primary antibodies and corresponding dilutions are shown in **Table S1,** secondary antibodies and corresponding dilutions are shown in **Table S2**.

Quantitative real time PCR of cultured cells

Total RNA was extracted using the total RNA purification kit (Norgen Biotek) following the manufacturer's instructions. cDNA synthesis was completed using the High-Capacity cDNA Reverse Transcription kit (Thermo Fisher). Quantitative real-time PCR was carried out using the QuantStudio 12 K Flex (Applied Biosystems) thermocycler with PowerUp SYBR master mix (Thermo Fisher) and the TaqMan Fast Universal PCR Master Mix (Applied Biosystems). Primer sequences and probe numbers where applicable are shown in Supplementary **Table S3**.

Single cell RNA sequencing and analysis

To create the gene expression matrix, we initially aligned the raw reads to the human genome (GRCh38) using Cell Ranger (10X Genomics, version 7.0.1) with its default settings. The resulting count data was imported into Scanpy (version 1.9.1), a single-cell analysis software. Quality control measures were applied by excluding cells expressing fewer than 200 genes and genes expressed in fewer than 3 cells. Additionally, cells with over 15% mitochondrial gene expression were removed. To detect and eliminate doublets, we used Scrublet with a

threshold of 0.25, and the estimated doublet ratio was determined as 0.14. For subsequent analyses, we combined the expression matrices, following the initial quality control steps, into a single Scanpy AnnData object containing 34,710 cells. Normalization and log transformation were performed using the default parameters. To mitigate the impact of cell proliferation, we removed cell cycle-related genes from the count matrix. The reference for cell cycle phase marker genes was obtained from the provided source (https://github.com/scverse/scanpy_usage/blob/master/180209_cell_cycle/data/regev_lab_cell_cycle_genes.txt). Next, we identified the top 3,000 highly variable genes and applied PCA dimension reduction to the data. We computed neighbors based on 35 nearest neighbors and 25 principal components. UMAP was generated with its default settings. We then focused on a subset of the dataset, specifically those from day 4 and day 6, and reanalyzed the subset separately. The re-clustering process used the same parameters as previously described, except for the neighbor graph calculation, which now used 10 neighbors and 10 principal components. Trajectory analysis was made using Scfates (2), the root was assigned to the cells expressing the gene NAV3. The cell cycle was detected by RNA velocity for S and G2M phase was calculated in scVelo (3). Additionally, we calculated the CytoTRACE score using the CellRank package (4) and RNA velocity using UniTVelo (5).

Fluorescence-activated cell sorting (FACS) isolation of ZsGreen positive ENS progenitors

At D21 post-transplant, surgical discard tissue segments transplanted with ENS progenitors (n=4 tissue segments) were washed in PBS, pooled and digested using a commercially available tumour dissociation kit (Miltenyi Biotec). Tissue was dissected into small pieces (~2mm^2) and incubated in the enzyme mix for 1 hour at 37°C, aspirating thoroughly to assist dissociation every 30 minutes. The resulting cell suspension was passed sequentially through a 70μm and a 40μm cell strainer. Cells were centrifuged and resuspended in FACS media (DMEM F12, Glutamine, HEPES (ThermoFisher Scientific) supplemented with N2

(Invitrogen), B27 (Invitrogen), Primocin (Invivogen) and 3% FBS (ThermoFisher Scientific). Cells were sorted based on ZsGreen fluorescence using a FACS AriaIII (BD Biosciences).

PCR analysis of FACS-isolated cells

RNA was extracted using an RNA mini kit (Qiagen, UK) according to the manufacturer's instructions, including optional treatment with DNase I (Qiagen). 100ng RNA was used to synthesise first-strand cDNA, using a SuperScript VILO cDNA Synthesis Kit (Life Technologies). Quantitative reverse transcriptase PCR (qRT-PCR) were conducted in a ABI Prism 7500 sequence detection system (Applied Biosystems) using a 96 well optical plate (Life Technologies) and the Quantitect SYBR Green PCR kit (Qiagen) according to the manufacturer's instructions. qRT–PCR was performed in triplicate using region-specific primers for *GAPDH, TuJ1*, *S100* and *SOX10* (**Supplementary Table 3**). Gene expression data were normalised to gene expression in D6 ENS progentiors (i.e., equivalent to those used at transplantation) using a 2^DCt calculation. For PCR, region-specific primers for *CALB2* and *NOS1* (**Supplementary Table 3**) were amplified using HotStarTaq DNA Polymerase (Qiagen) in a T100 Thermocycler (BioRad). The amplification profile used was as follows: 94°C (3min), 35 cycles of 94°C (30s), 60°C (60s) and 72°C (30s), followed by a final step of 72 °C (2 min). Products were re-amplified using the same amplification profile and the products analysed on a 2% agarose gel alongside a 25 bp Hyperladder (Bioline).

Quantification of transplanted cell migration

For a subset of transplants (n=4 transplanted segments) images were captured at 7-day intervals through *ex vivo* culture to capture cell migration. Magnification, exposure and gain were kept constant throughout. These images were then processed using FIJI and the following, custom-designed Macro:

```
{namelong = getTitle();
name = substring(namelong, 0, lengthOf(namelong)-4);
```

```
run("Duplicate...", "title=Ch2 duplicate channels=2");
run("Duplicate...", "title=Ch2spots");
run("Remove Outliers...", "radius=5 threshold=20 which=Bright");
imageCalculator("Difference create", "Ch2","Ch2spots");
setAutoThreshold("Triangle dark");
run("Convert to Mask");
rename("Mask");

imageCalculator("AND create", "Mask","Ch2");
rename(name+"-OutlierProcessed");
selectImage("Mask");
rename(name+"-OutlierMask");

close("*Ch2*");}
```

Tissue clearing: patient-derived HSCR colonic surgical discard tissue

Transplanted and sham-transplanted tissue explants were fixed in formaldehyde (PFA, 4% w/v) for 1hr at RT and washed in PBS. Samples were blocked with 1% bovine serum albumin (Sigma Aldrich) and 1% Triton X-100 (Sigma Aldrich) in PBS at RT before incubation in primary antibody (Table S1) diluted in blocking solution for 72 hours at 4°C. Secondary antibodies (Table S2) diluted in blocking solution were applied for 48 hours at 4°C. To visualise transplanted ENS progenitors, transplanted tissue segments were cleared using FluoClearBABB (6). Segments were dehydrated in an ascending series of tert-butanol (Thermo Fisher Scientific Inc.), buffered to pH9.5 using triethylamine) and incubated overnight at RT in 100% tert-butanol (pH9.5, Thermo Fisher Scientific Inc.). Samples were incubated in a 1:1 ratio of tert-butanol:BABB (1:2 benzoic acid:benzyl benzoate) for 1 hour at RT, transferred to 100% BABB and stored at 4°C until imaging. Prior to imaging, samples were transferred to ethyl cinnamate (Sigma Aldrich). Immunostained and cleared tissue segments

were imaged using an LSM710 (Zeiss), LSM 880 with Airyscan (Zeiss) and an AxL Cleared Tissue LightSheet (3I).

SUPPLEMENTARY TABLES

Table S1: List of primary antibodies used in this study

Antibody	Cat no.	Company	Dilution used	Application
OCT4	SC5279	Santa Cruz	1:250	Flow cytometry
NANOG	D7364	Cell Signalling Technologies	1:400	Flow cytometry
SOX10	D5V9L	Cell Signalling Technologies	1:500	Flow cytometry
TUBB3	ab78078	Abcam	1:1000	IF (*in vitro* neuronal cultures)
RET[EPR2871]	ab134100	Abcam	1:1000	IF (*in vitro* neuronal cultures)
PGP9.5	ab108986	Abcam	1:1000	IF (*in vitro* neuronal cultures)
Peripherin	AB1530	Millipore	1:500	IF (*in vitro* neuronal cultures)
nNOS	61-7000	Invitrogen	1:400	IF (*in vitro* neuronal cultures)
HuC/HuD	A-21271	Invitrogen	1:200	IF (*in vitro* neuronal cultures)
S100β	S2532	Sigma	1:1000	IF (*in vitro* neuronal cultures)

CD49d	304302	BioLegend	1:100	IF (*in vitro* neuronal cultures)
TUBB3	801202	Biolegend	1:500	IF (wholemount tissue)
S100β	ab52642	Abcam	1:100	IF (wholemount tissue)

Table S2: List of secondary antibodies used in this study

**Antibody**	**Cat No.**	**Company**	**Dilution used**	**Application**
Alexa Fluor® 488	A-21206	Invitrogen	1:500	IF, Flow cytometry
Alexa Fluor® 568	A-11004	Invitrogen	1:500	IF
DAPI	D9542	Sigma	1:1000	IF
Alexa Fluor® 594	A-21203	Invitrogen	1:500	IF (*in vitro* neuronal cultures)
Alexa Fluor® 647	A-31573	Invitrogen	1:500	IF (*in vitro* neuronal cultures)
Alexa Fluor® 488	A-21202	Invitrogen	1:500	IF (*in vitro* neuronal cultures)

Table S3: List of primers used in this study

qRT-PCR primers used			
**Gene**	**Direction**	**Sequence (5' - 3')**	**Probe (where applicable)**
HOXB4	F	AAACCAGGCCCCTTCCTAC	
	R	GCACACAGATATTCACACATACGA	
HOXB5	F	AAGCTTCACATCAGCCATGA	
	R	CGGTTGAAGTGGAACTCCTTT	
HOXB7	F	CTACCCCTGGATGCGAAG	
	R	CAGGTAGCGATTGTAGTGAAATTCT	
SOX10	F	TTGCTGCCTCTTTAAGACTAGGA	
	R	TAAGCCTGGGGCTCAAACT	
PAX6	F	CGAGATTTCAGAGCCCCATA	
	R	AAGACACCACCGAGCTGATT	
GAPDH	F	CCCCGGTTTCTATAAATTGAGC	60
	R	CTTCCCCATGGTGTCTGA	
GAPDH	F	GATTCCACCCATGGCAAAT	
	R	TGGACTCCACGACGTACTCA	
TUBB3	F	CATTCTGGTGGACCTGGAAC	
	R	CCTCCGTGTAGTGACCCTTG	
S100	F	GAAGGGAGGGAGACAAGCAC	
	R	TCCTGGAAGTCACATTCGCC	
CALB2	F	GCGATCTTCACATTTTACGACA	42
	R	TCATTTCCTTTTTGTTTTTCTCG	
NOS1	F	TGAGTCCATTGCGTTCATTG	
	R	TCCGCGCTTACAAAACTTG	

TH	F	ACGCCAAGGACAAGCTCA	42
	R	AGCGTGTACGGGTCGAACT	
Peripherin	F	AAGACGACTGTGCCTGAGGT	10
	R	TGCTCCTTCTGGGACTCTGT	
TRKC	F	CCGTACGAGAGGGTGACAAT	21
	R	TGGTCCAGTTCAGATTGGTCT	
RET	F	CTCCCTTCCACATGGATTGA	80
	R	TCAGCTCTCGTGAGTGGTA	

References:

1. Schindelin J, Arganda-Carreras I, Frise E, Kaynig V, Longair M, Pietzsch T, et al. Fiji: an open-source platform for biological-image analysis. Nat Methods. 2012;9(7):676-82.
2. Faure L, Soldatov R, Kharchenko PV, Adameyko I. scFates: a scalable python package for advanced pseudotime and bifurcation analysis from single-cell data. Bioinformatics. 2022;39(1).
3. Bergen V, Lange M, Peidli S, Wolf FA, Theis FJ. Generalizing RNA velocity to transient cell states through dynamical modeling. Nat Biotechnol. 2020;38(12):1408-14.
4. Gulati GS, Sikandar SS, Wesche DJ, Manjunath A, Bharadwaj A, Berger MJ, et al. Single-cell transcriptional diversity is a hallmark of developmental potential. Science. 2020;367(6476):405-11.
5. Gao M, Qiao C, Huang Y. UniTVelo: temporally unified RNA velocity reinforces single-cell trajectory inference. Nature Communications. 2022;13(1):6586.
6. Schwarz MK, Scherbarth A, Sprengel R, Engelhardt J, Theer P, Giese G. Fluorescent-protein stabilization and high-resolution imaging of cleared, intact mouse brains. PLoS One. 2015;10(5):e0124650.
