# Supplementary figures and images for: Human enteric nervous system progenitor transplantation improves functional responses in Hirschsprung disease patient-derived tissue
Source: Gut. 2024 May 30;73(9):1441–53. doi: 10.1136/gutjnl-2023-331532 (PMC11347211; doi:10.1136/gutjnl-2023-331532)

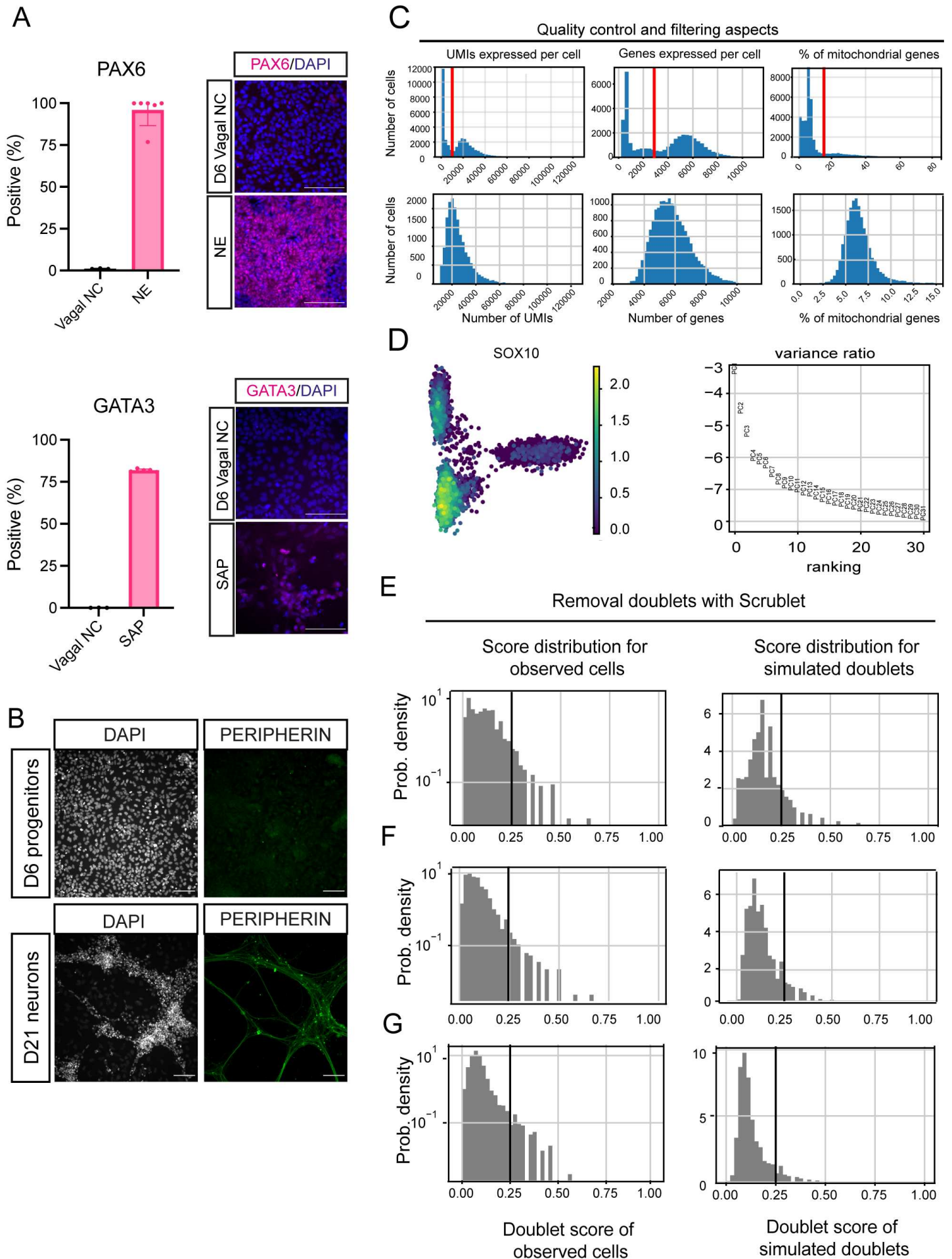

Supplement: Supplementary data [file gutjnl-2023-331532supp001.pdf]

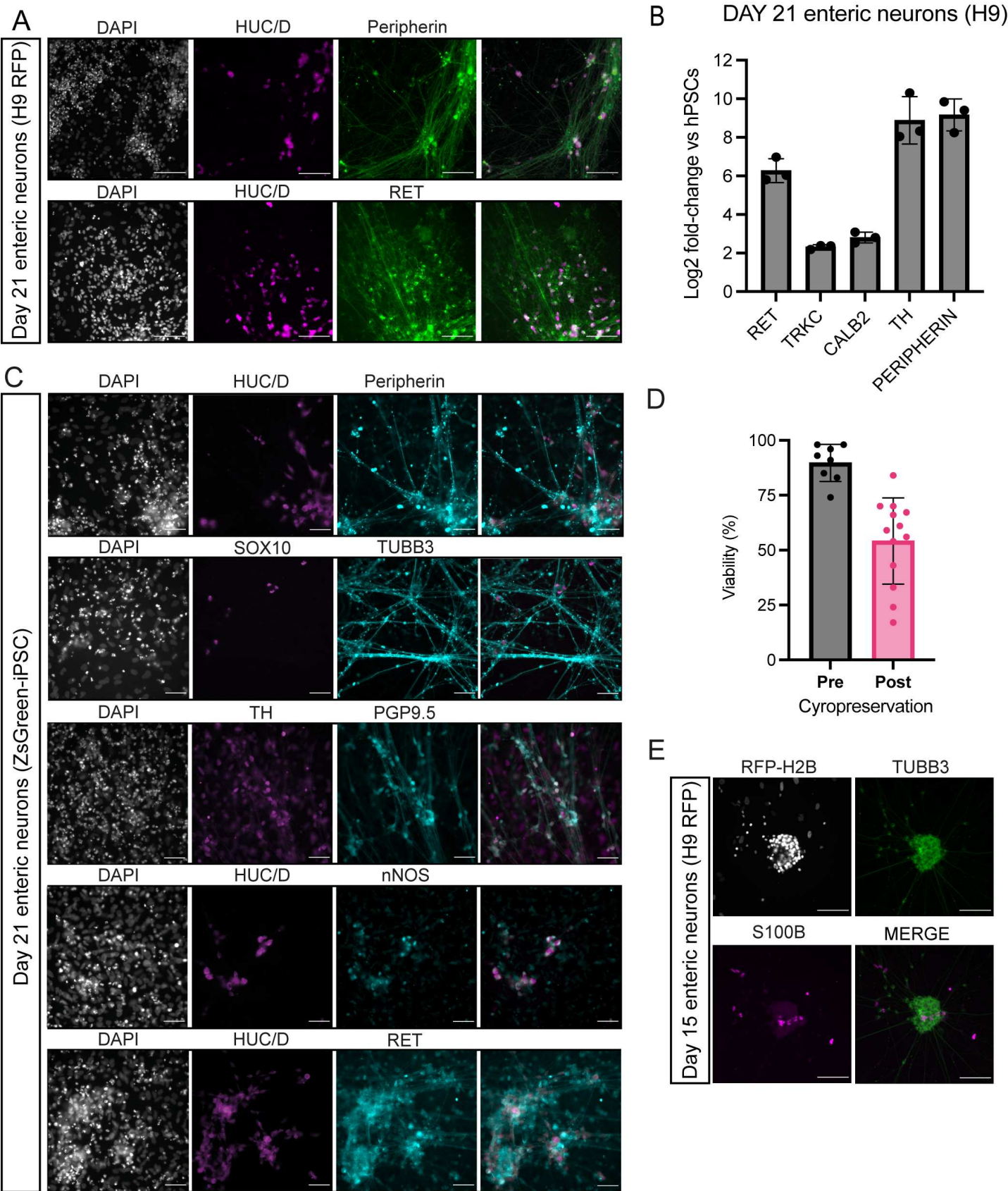

Supplement: Supplementary data [file gutjnl-2023-331532supp002.pdf]

## 3D representation of cell integration

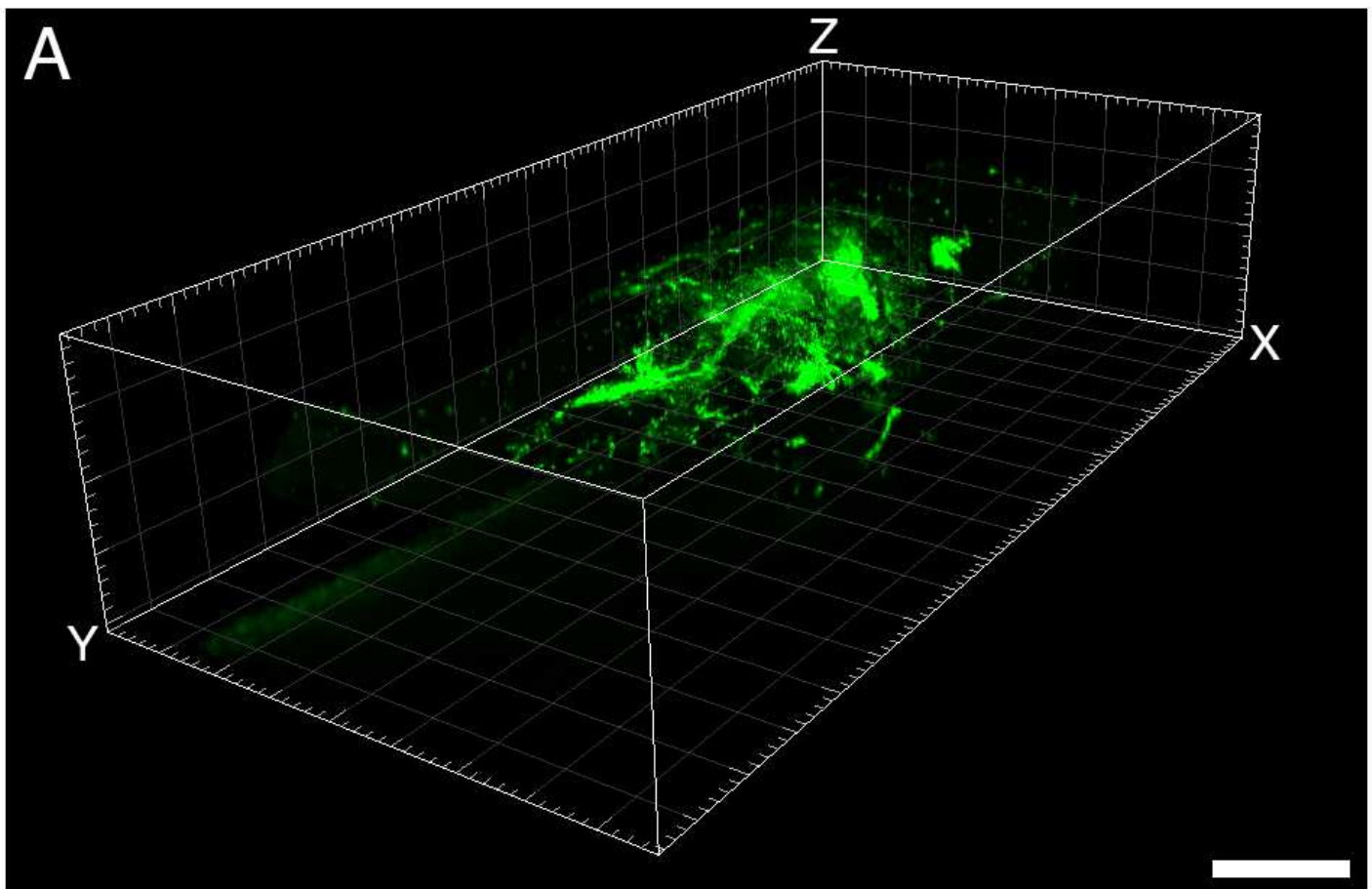

Supplement: Supplementary data [file gutjnl-2023-331532supp003.pdf]

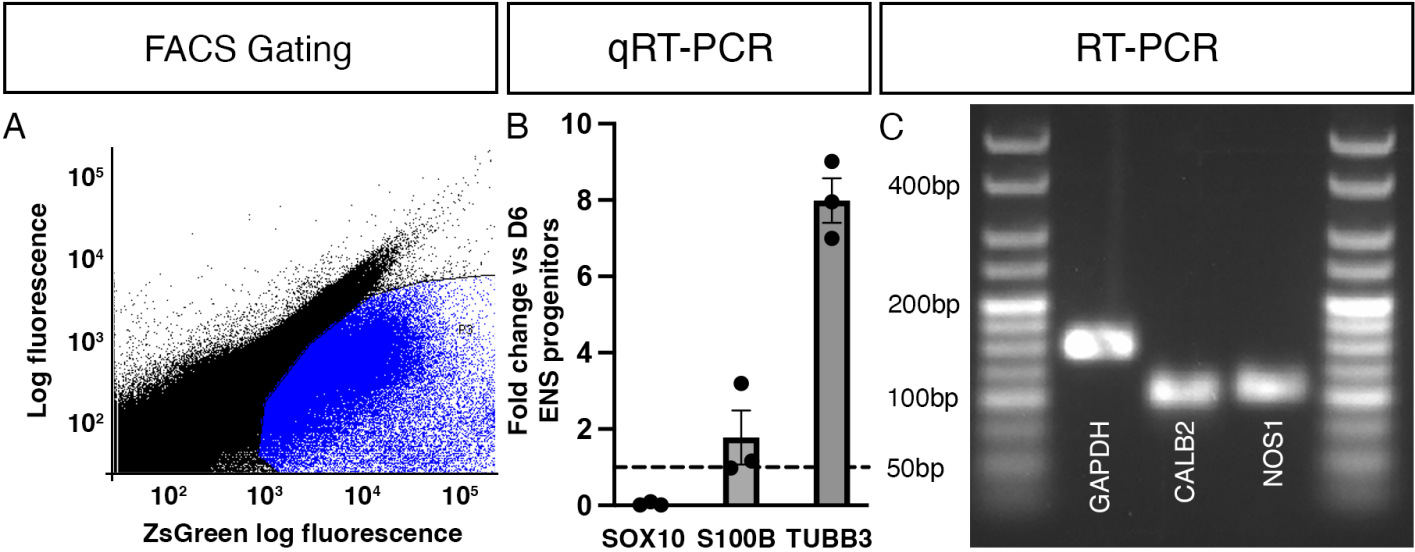

Supplement: Supplementary data [file gutjnl-2023-331532supp004.pdf]
